# Efficacy of concentrated growth factor versus collagen membrane in reconstructive surgical therapy of peri-implantitis: 3-year results of a randomized clinical trial

**DOI:** 10.1007/s00784-022-04493-y

**Published:** 2022-05-26

**Authors:** Sila Cagri Isler, Fatma Soysal, Tugce Ceyhanlı, Batuhan Bakırarar, Berrin Unsal

**Affiliations:** 1 Department of Periodontology, Faculty of Dentistry, Gazi University, Ankara, Turkey; 2 Department of Periodontology, School of Dental Medicine, University of Bern, Freiburgstrasse 7, CH-3010 Bern, Switzerland; 3 Department of Periodontology, Faculty of Dentistry, Ankara Medipol University, Ankara, Turkey; 4 Department of Periodontology, Faculty of Dentistry, Yeditepe University, Istanbul, Turkey; 5 Department of Biostatistics, Faculty of Medicine, Ankara University, Ankara, Turkey

**Keywords:** Peri-implantitis, Membranes, Blood platelets, Reconstructive surgical procedures, Submerged healing, Maintenance

## Abstract

**Objectives:**

To compare the 3-year clinical and radiographic outcomes of two different reconstructive surgical management of peri-implantitis using a bone substitute in combination with either concentrated growth factor (CGF) or collagen membrane (CM).

**Material and methods:**

Fifty-one patients who had at least one implant presenting peri-implantitis with an intrabony defect were filled with a xenogenic bone grafting material and covered either CGF or CM. Clinical and radiographic assessments were carried out at baseline and postoperative years 1 and 3. Three different composite outcomes were defined to evaluate treatment success at a 3-year follow-up. The effects of possible prognostic indicators on treatment success were identified by using multilevel regression analysis.

**Results:**

The changes in probing depth (PD) and radiographic vertical defect depth (VDD) between baseline and year 1 and baseline and year 3 presented significantly greater decreases for the CM group in comparison with the CGF group (*p* < 0.05). No significant differences between the two treatment modalities were demonstrated regarding treatment success outcomes. History of periodontitis, VDD at baseline, and the number of intrabony defect walls revealed significant impacts on treatment success (*p* = 0.033; OR = 3.50, *p* = 0.039; OR = 0.975, and *p* = 0.024; OR = 7.0 and *p* = 0.019;OR = 6.0, respectively).

**Conclusions:**

CM in combination with a bone substitute seems to have slightly better outcomes compared to the CGF membranes in reconstructive surgical therapy of peri-implantitis. The history of periodontitis, baseline VDD, and peri-implant bone defect configuration could be possible predictors influencing treatment success.

**Trial registration:**

ClinicalTrials.gov NCT04769609.

**Clinical relevance:**

For the reconstruction of peri-implant bone defects, using a bone substitute in combination with a collagen membrane may show more favorable outcomes.

## Introductıon

Peri-implantitis is a plaque-associated pathological disease that affects the tissues surrounding a dental implant and is clinically characterized by bleeding on probing (BOP) and/or suppuration (Supp), increased probing depths (PDs), and/or mucosal marginal recessions (MRs) in addition to progressive supporting bone loss [1]. Recent data has revealed that it presents the most common etiological factor for late implant loss [2]. Considering the increase in the number of patients undergoing restorative treatment through dental implants and accordingly the increments in the prevalence of peri-implantitis (15–57% at the patient level and 8–28% at the implant level) [3], it is imperative to apply effective treatment methods to manage these conditions. Otherwise, if peri-implantitis lesions are left untreated, they progress in a non-linear and accelerating manner [1, 4]. Hence, peri-implantitis has been considered a major, unpredictable and growing problem for clinicians.

Various treatment protocols for this serious problem involve non-surgical approaches and surgical therapies with open flap debridement procedures, resective surgeries, or reconstructive modalities, which include the use of bone substitutes with or without a membrane, decontamination methods of implant surfaces, antimicrobial agents in the principles of non-submerged or submerged approaches, have been proposed [5]. Although non-surgical interventions have been reported to be effective in reducing BOP and PDs in peri-implantitis sites, these treatments have appeared to be unpredictable for the management of peri-implantitis owing to the fact that they allow limited access to the contaminated and nonshedding implant surface [6–8]. Surgical non-reconstructive approaches, i.e., open-flap debridement, which provide a direct vision for implant surface decontamination, have been reported to improve peri-implant tissue health and maintain stable marginal bone levels [8]. However, this surgical approach has favorable outcomes, particularly in the presence of supracrestal peri-implant defects and limited efficacy in relieving inflammation in the long term accompanied with significant postoperative soft-tissue recession [8, 9]. For the management of peri-implantitis cases exhibiting intrabony defects and/or more advanced lesions, surgical augmentative approaches have been suggested to be performed to get more predictable clinical and radiographical outcomes [8, 10]. Several surgical augmentative therapy studies, including the use of bone substitutes with or without barrier membranes, have demonstrated significant clinical and radiographic improvements for at least 3 years, especially in well-contained (three- or four-wall) intrabony defects [9, 11–13]. On the other hand, no clear evidence has been found to support the superiority of a specific material, product, or membrane in long-term clinical outcomes of a reconstructive therapy [1, 8]. It has been strictly recommended that identification of peri-implantitis treatment success and disease resolution in the long-term management are required to allow adequate assessment of stable treatment outcomes.

Because of many studies indicating that growth factors (GFs) enable to transiently stimulate cells locally, accelerate angiogenesis, and promote proliferation, differentiation, and regeneration, the additional use of them in the management of peri-implantitis has been proposed to improve clinical outcomes and enhance soft and hard tissue regeneration [14, 15]. A recent systematic review and meta-analysis have suggested that GFs, including enamel matrix derivates and autologous platelet concentrates, might be associated with better outcomes with regard to PD and BOP whereas they have not revealed statistically significant evidence for any additional benefit in peri-implantitis treatment [14]. Concentrated growth factor (CGF) is an autologous platelet concentrate product that is characterized by containing plentiful GFs including bone morphogenetic protein-2 (BMP-2) within its considerably rigid fibrin in relation to a different centrifugation speed protocol compared to advanced platelet-rich fibrin (A-PRF) [16]. It has been suggested that CGF provides a strong biological scaffold and acts as an integrated reservoir for the slow release of closely interconnected GFs, thereby helping to accelerate tissue regeneration [17]. Nonetheless, there is hardly any information in the literature regarding the predictability and long-term stability of platelet concentrates in peri-implantitis management.

It was, therefore, the purpose of the present study to analyze the 3-year clinical/radiographic outcomes of reconstructive surgical treatment of peri-implantitis by means of a bone substitute in combination with two different bioresorbable barrier membranes, either CGF or collagen membrane (CM), and also to identify prognostic indicators influencing the long-term reconstructive surgical treatment outcome, using a multilevel statistical model.

## Material and methods

### Study design and patient population

**Fig. 1:**
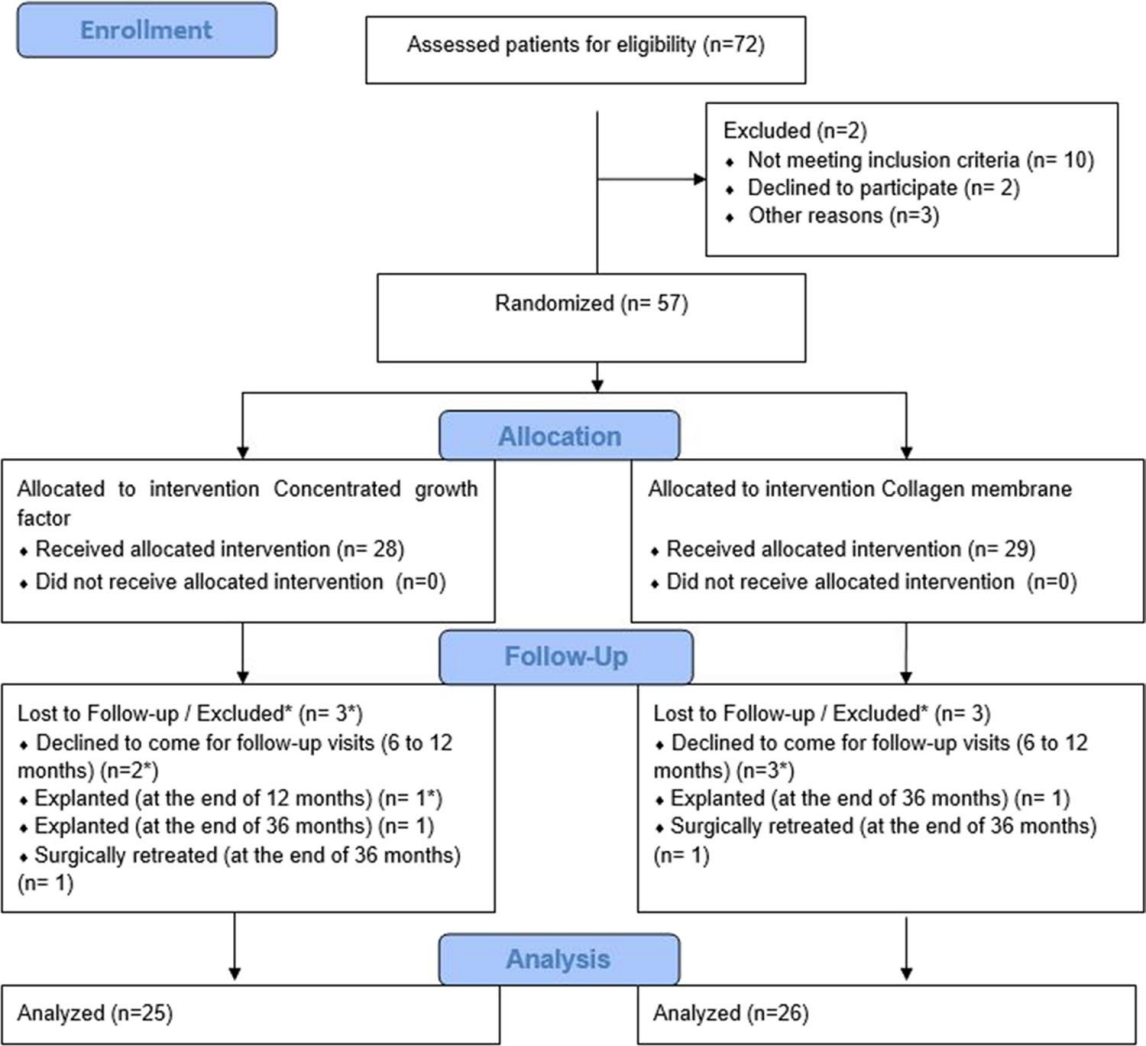
The CONSORT flow chart of the study

This study was a prospective observational study with the registration of clinical and radiographic outcomes at 1 and 3 years after a reconstructive surgical therapy of peri-implantitis. The study design was approved by the Clinical Research Ethics Committee of Gazi University, Faculty of Dentistry (GUDHKAEK.2021.01/4), and the study was conducted according to the principles outlined in the Declaration of Helsinki and Ethical Conduct for Research with Human Beings and the Good Clinical Practice Guidelines. The trial was registered at ClinicalTrials.gov (NCT04769609). The study protocol is in compliance with the CONSORT guidelines and the checklist is annexed as File S1. The study was carried out on a patient population from a previously published RCT [18] between February 2015 and February 2018, and afterwards, the patients were enrolled in supportive peri-implant/periodontal therapy (SPIT) until March 2021. Each patient was given a detailed description of the study procedures and a written informed consent prior to being included in the study. Study participants were recruited from among the subjects referred to the Department of Periodontology, Faculty of Dentistry, Gazi University, for treatment of peri-implant disease. A total of 72 patients who had at least one implant diagnosed with peri-implantitis and needed to be scheduled for reconstructive therapy of a peri-implant infrabony defect were included. Figure 1 depicts the flowchart of the study.

The study protocol was previously described in detail [18]. Briefly, in the present study protocol, peri-implantitis case definition was derived from the consensus from the 8^th^ European Workshop on Periodontology [19]. The patients having at least one implant demonstrating two-, three-, or four-wall infrabony defects ≥ 3 mm, which presented a PD of ≥ 5 mm with BOP and/or suppuration were included in the study. The exclusion criteria were defined as the presence of serious systemic diseases, medications, or conditions that were contraindicated for periodontal surgery and would compromise wound healing; a history of taking systemic antibiotic over the past 3 months; placement and prosthetic loading of implants within the past year; and presence of one-wall peri-implant intrabony defects, implants with prosthetic suprastructure that were impossible to remove, implant mobility, or evidence of occlusal overload.

The study participants received reconstructive surgical treatment using a bone substitute combined with either CGF (CGF group) or CM (CM group). Subjects were grouped by a permuted block design with a computer random-number generator (allocation ratio of 1:1). Group allocation was concealed in an envelope with identification codes, which were opened immediately before the placement of bone grafts and barrier membranes to the defects. Treatment assignment for each patient was registered by the clinician who assisted the operations, and such assignment was kept concealed until the end of the study.

### Surgical intervention and post-surgical protocol

All patients underwent an oral hygiene program and received a non-surgical therapy consisting of supra and subgingival/mucosal mechanical debridement for their teeth and implants to resolve the inflammation (i.e., suppuration and pus formation) 4–6 weeks before the surgical intervention. No surgery was performed before all periodontal treatments were finished along with the patient’s compliance had been verified as good. Before proceeding with the surgical treatment, the prosthetic suprastructures were removed and cover screws were placed to increase soft tissue coverage so that guided bone reconstruction can be provided in a submerged manner. Implants were submerged for a postoperative 6-month period, then suprastructures were replaced after healing.

**Fig. 2:**
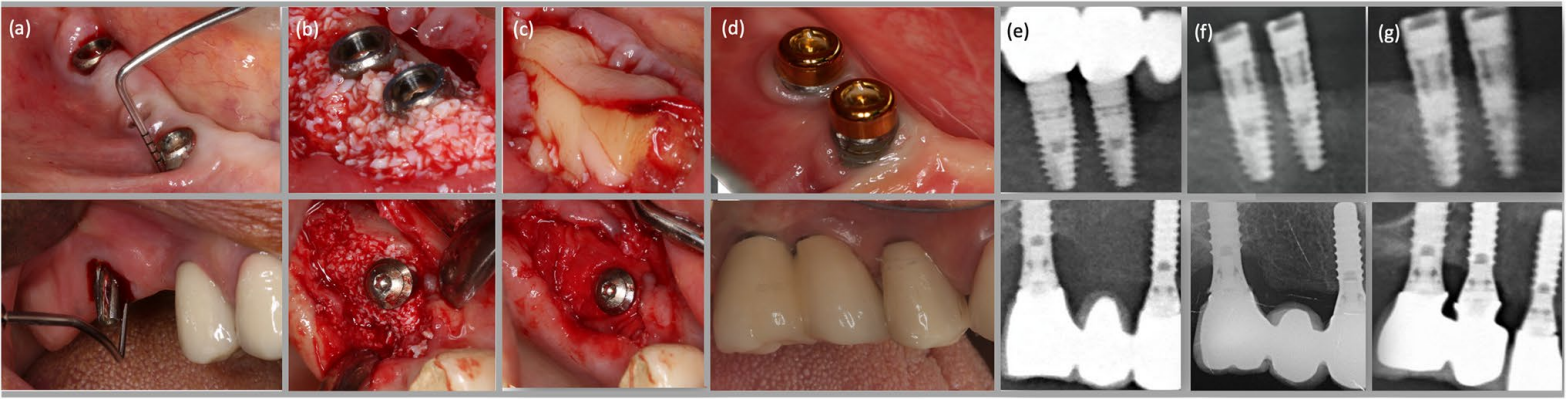
Representative clinical and radiographic outcomes following reconstructive therapies at 3 years. **a** Baseline clinical situation presenting excessive PD with BOP and Supp. **b** Filling of the infrabony component of the defect with a bovine-derived xenogenic particulate graft material. **c** Covering of the entire defect with either CGF or collagen membrane. **d** Clinical situations at the 3-year follow-up presenting no signs of inflammation and minimal PD. **e** Baseline radiographs. **f** Radiographic images at the 1-year follow-up. **g** Radiographic images at the 3-year follow-up

All surgeries were performed by two experienced periodontists (B.U. and S.C.I.). Sulcular incisions were made around the neck of the implants that were extended mesially and distally to raise full-thickness mucoperiosteal flaps at the buccal and lingual aspects. Inflammatory tissues were completely removed from the defect, and implant surfaces were debrided by titanium curettes (ImplaMate, Nordent Mfg Inc., Elk Grove Village, IL). The surfaces were then irrigated with saline solution (20 mL, 20 s). Then the intrabony defects in both study groups were filled with a xenogenic particulate graft material (Bio-Oss spongiosa granules, particle size 0.25–1 mm; Geistlich Biomaterials, Wolhusen, Switzerland). For the preparation of CGF, 10 mL glass-coated plastic tubes (Vacutainer tube® BD, Franklin Lakes, NJ, USA) were used to collect blood samples, and then they were immediately centrifuged utilizing a centrifugation device (Medifuge, Silfradent, S. Sofia, Italy) with a 33° rotor angulation and with a radius of 50 mm at the clot. CGF membranes were produced utilizing a protocol as follows: 30″ acceleration, 2′ 2700 rpm, 4′ 2400 rpm, 4′ 2700 rpm, 3′ 3000 rpm and 36″ deceleration and stop (RCF-clot = 2′, 692 g; 4′, 547 g; 4′, 592 g; 3′, 855 g). Two pieces of CGF membranes were placed over the graft material and adapted to the entire defect in the CGF group. In the CM group the same graft material was covered with a bioresorbable collagen membrane (Bio-Guide, Geistlich Biomaterials) (Fig. 2). Then, the flaps were repositioned coronally and stabilized without any tension using a 4–0 nonresorbable suture (Dogsan Surgical Sutures, Trabzon, Turkey).

Peri-implant bone defect configurations were evaluated at the time of surgical intervention on the basis of the number of residual bone walls as two-, three-, and four-wall intrabony defects.

Post-surgically, the patients were recommended to use a 0.12% CHX mouth rinse twice a day for two weeks. The antibiotic protocol included administering 500 mg amoxicillin and 500 mg metronidazole three times a day for 1 week. Anti-inflammatory and analgesic drugs (flurbiprofen 100 mg, twice a day) were prescribed during the first 3 days after the surgery. The sutures were removed approximately 2 weeks after the surgery.

### Supportive peri-implant therapy

All the participants enrolled in a maintenance program following the surgical interventions and received supportive peri-implant therapy (SPIT). Follow-up examinations were performed every third month from 6 to 36 months after the reconstructive surgical treatments of peri-implantitis. At each appointment, supragingival/mucosal mechanical debridement and reinforcement of oral hygiene aiming for a low full mouth plaque score were provided. When necessary, localized subgingival/mucosal instrumentation in combination with pocket irrigation using saline solution was done except for the surgical area until 1-year postoperatively. Later, individual supportive care and non-surgical approaches were applied at signs of recurrence (increasing PD with concomitant BOP compared to the previous examinations) in the whole mouth every 3–6 months according to the patient’s risk profiling for three years postoperatively [20].

### Clinical and radiographic assessments

A calibrated single examiner (F.S.) who was not involved in the surgical procedure was responsible for all clinical parameters. The following clinical measurements were evaluated using a manual periodontal probe (N116, Nordent Mfg Inc., Elk Grove Village, IL) on four implant sites (mesial, buccal, distal, and palatal/lingual) at baseline and 1-year and 3-year follow-ups: Plaque index (PI) [21], Gingival index (GI) [22], PD, BOP, Supp, MR, and Clinical attachment level (CAL).

Another blinded and calibrated examiner (T.C.) measured the mesial and distal radiographic marginal bone level at baseline and 1 year and 3 years postoperatively of the treated implants according to the procedure previously described [18]. In short, at the mesial and the distal aspects of each implant, the distance between the first bone-to-implant contact and a well-defined reference point in the coronal portion of implant body was measured in millimeters by means of the periapical radiographs, which were obtained with the long-cone paralleling technique with an individualized film holder (Rinn bite film holder for periapical radiographs, Dentsply, York, PA). The vertical defect depth (VDD) measurement was provided by taking the average of both mesial and distal measurements. The radiographic reduction of the intrabony component named radiographic defect fill (DF) was evaluated as the VDD difference between baseline and follow-up examinations 1 year and 3 years after the reconstructive surgery.

### Treatment outcomes

A composite outcome, which was also considered a determinant of treatment success, was used to determine whether an implant was successfully treated without requiring any further surgical intervention [23]. The proposed success criteria were categorized as follows:

- Outcome 1: the absence of additional bone loss after the treatments according to baseline radiographs;
- Outcome 2: absence of additional bone loss with a maximum PD depth of ≤ 5 mm;
- Outcome 3: absence of additional bone loss with a maximum PD depth of ≤ 5 mm, and without any BOP or Supp [24].

### Statistical analysis

The sample size was calculated by a mean radiographic defect depth (VDD) in 3 years (2.77 ± 1.56 and 1.99 ± 1.43, respectively) with α = 0.05 and the power of 80% using the Student t test. Based on this calculation, 17 subjects were found to be required for each group.

For each patient, one implant with the most severe peri-implant defect was taken as a statistical unit. Clinical and radiographic quantitative variables were expressed as mean values (± SD) and median (min–max), and categorical variables as number (*n*) and percentage (%). The Kolmogorov–Smirnov test was used to assess the normality of distributions. Comparison of quantitative variables was calculated using Student’s t test or Mann–Whitney U test, whereas Pearson chi-square or Fisher exact test were used for qualitative variables. A generalized linear mixed model (GLMM) was used to compare mean differences between groups that included between-subjects and within-subjects factors in terms of time trend (increase and decrease). Within groups, repeated measures of ANOVA were used to show the significance for each group. When a significant difference was found based on the time within groups, pairwise comparisons were made to find out which two different time intervals caused the difference. Independent Student’s t-test was performed to compare treatment outcomes between the groups. A multivariate logistic regression test was used in order to investigate the influence of patient demographics, implant characteristics, and baseline clinical and radiographic parameters in addition to treatment methods on the composite outcome. *p* < 0.05 was accepted for the significance level of the tests.

## Results

Of the initial 72 patients, who met the inclusion criteria for the study, 51 patients completed the 3-year follow-up examination. During the first year, 2 patients from the CGF group and 3 patients from the CM group did not attend follow-up appointments properly. One implant with progressive bone loss concomitant with Supp and increased PD in the CGF group had to be explanted between the 1- and 3-year follow-ups. Thus, these patients were dropped out from the current study (3 patients (10.7%) in the GGF group, and 3 patients (10.3%) in the CM group). The patients, who fully adhered to SPIT were included in the final analysis. At the end of the 3-year follow-up examination, non-reconstructive surgical therapy, including open flap debridement, was performed on one implant from the CM group, and two implants from the CGF group due to the severe peri-implant inflammation accompanying Supp after their final assessments (Fig. 1).

Patient demographics, implant and site-level characteristics, and the distribution of peri-implant bone defect types were reported in Table 1. The mean age was 57.88 ± 9.24 years in the CGF group, 56.15 ± 9.23 years in the CM group. Eleven patients (44.0%) belonging to the CGF group had a history of periodontitis, while 13 patients (50%) had a history of periodontitis in the CM group. Regarding postoperative complications, three implants from the CM group (11.5%) were exhibited a slight membrane exposure without signs of inflammation, whereas no complication was observed for the CGF group during the submerged healing. All cases resulted in almost complete soft tissue coverage within 2–3 weeks postoperatively.

**Table 1:** Demographic data on patients and affected implants characteristics

	Total	CGF group	CM group
Age, mean (SD)	57.0 (9.18)	57.88 (9.24)	56.15 (9.23)
Years of implant in function, mean (SD)	4.93 (2.10)	4.65 (1.63)	5.21 (2.48)
Gender Male, *n*(%) Female, *n*(%)	27 (52.94) 24 (47.05)	16 (64.00) 9 (36.00)	11 (42.30) 15 (57.69)
Smoking, *n*(%)	15 (29.41)	6 (24.00)	9 (34.62)
History of periodontitis, *n*(%)	24 (47.05)	11 (44.00)	13 (50.00)
Implant surface category Non-modified, *n*(%) Modified, *n*(%)	10 (19.60) 41 (80.39)	5 (20.00) 20 (80.00)	5 (19.23) 21 (80.76)
Implant position Maxilla, *n*(%) Mandible,*n*(%) Anterior, *n*(%) Posterior, *n*(%)	21 (41.17) 31 (60.78) 6 (11.76) 45 (88.23)	13 (52.00) 12 (48.00) 3 (12.00) 22 (88.00)	7 (26.92) 19 (73.07) 3 (11.53) 23 (88.46)
Type of restoration Fixed partial denture, *n*(%) Single crown, *n*(%)	39 (76.47) 12 (23.52)	20 (80.00) 5 (20.00)	19 (73.07) 7 (26.92)
Number of peri-implant intrabony defect walls Two-wall Three-wall Four-wall	22 (43.14) 12 (23.52) 17 (33.33)	11 (44.00) 5 (20.00) 9 (36.00)	11 (42.30) 7 (26.92) 8 (33.78)

*CGF*, concentrated growth factor; *CM*, collagen membrane

At the 3-year follow-up, 5 implants (20%) were diagnosed as healthy, 11 implants (40%) had peri-implant mucositis, and 9 implants (36%) were diagnosed with a recurrence of peri-implantitis in the CGF group. However, in the CM group, 9 implants (34.6%) were diagnosed as healthy, 12 implants (46.2%) had peri-implant mucositis, and 5 implants (19.2%) showed recurrence of peri-implantitis.

**Fig. 3:**
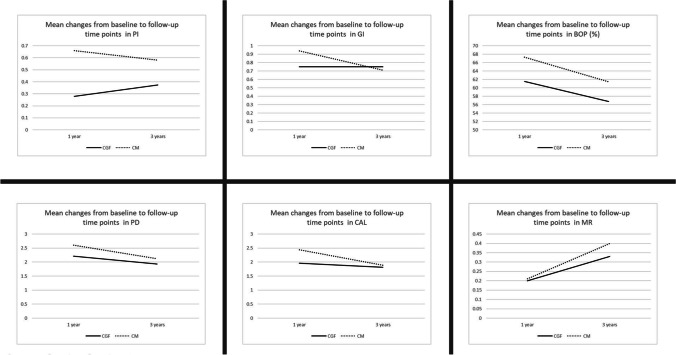
Mean changes in clinical parameters at 1 and 3 years postoperatively. CGF, Concentrated growth factor; CM, Collagen membrane; PI, plaque index; GI, gingival index; BOP, bleeding on probing; PD, probing depth; CAL, clinical attachment level; MR, mucosal recession. Mann–Whitney test, *p* < 0.05 considered statistically significant

An overall improvement was observed according to the baseline conditions with respect to mean PI, GI, and BOP values during the 3-year study time periods (Table 2). The mean PI reduced significantly to 0.67 ± 0.37 at year 1, and 0.59 ± 0.50 at year 3 in the CGF group, while in the CM group, the same variable was found around 0.45 ± 0.43 at year 1 and 0.52 ± 0.45 at year 3. A similar trend was also observed for the mean GI values (0.36 ± 0.45 and 0.13 ± 0.29) at year 1 and (0.37 ± 0.54 and 0.32 ± 0.49) at year 3 in the CGF and CM groups, respectively. In the CGF group, the mean BOP declined from 97.12 ± 10.79 to 35.58 ± 30.14% at year 1, and to 40.38 ± 33.22% at year 3, while in the CM group, it was decreased from 97.12 ± 8.15 to 29.81 ± 30.02% at year 1, and to 35.58 ± 33.30% at year 3. Eight patients (30.7%) in the CGF group and nine patients (34.6%) in the CM group showed a complete resolution of inflammation without presenting any BOP and Supp at the 3-year examination. The mean PD was 5.92 ± 1.26 mm and 5.41 ± 1.16 mm at baseline in the CGF and CM groups, respectively, without any significant differences between them. Comparing the trend of decreases at the follow-up periods based on baseline values belonging to the study groups, a trend towards a significantly higher reduction was observed in the CM group compared to that in the CGF group (*p* = 0.007). A trend of increase was seen at year 3 (CGF group, 3.80 ± 1.41 mm; CM group, 3.28 ± 1.27 mm) compared to year 1 postoperatively (CGF group, 3.71 ± 1.09 mm; CM group, 2.70 ± 0.80 mm) for both groups, and the increase was found statistically significant for the CM group (*p* = 0.005); however, it did not reach statistical significance in the CGF group (*p* = 0.582). In line with PD, while a significant decrease was observed at year 1 and year 3 compared to the baseline for the mean CAL values in both groups, it could be observed that the third-year values tended to increase compared to the first year values. Besides, the changes over time in CAL did not reveal any statistically significant difference between the groups. The mean MR at baseline was 0.04 ± 0.20 mm and 0.06 ± 0.20 mm for tthe CGF and CM groups, respectively. In both groups, increases were identified for the mean recession levels at year 1 and year 3 compared to the baseline (0.25 ± 0.39 mm and 0.27 ± 0.44 mm at the year 1 follow-up; 0.38 ± 0.48 mm and 0.46 ± 0.55 mm at the year 3 follow-up, respectively). Although the increases in the CGF group were lower than the CM group in the follow-up examinations, there were not any significant differences between the two groups in terms of the changes over time. The mean changes at year 1 and year 3 follow-ups for PI, GI, BOP, PD, and CAL and during the course of the study are presented in Fig. 3.

**Table 2:** Comparison of the mean clinical and radiographic parameters measured at baseline and 1 year and 3 years

Parameters		CGF (*n* = 25)		*p* value between time points^†^	CM (***n*** = 26)		*p* value between time points^†^	*p* value^*^
		Mean ± SD	Median (min–max)		Mean ± SD	Median (min–max)		
Plaque index	Baseline	0.96 ± 0.89	1.00 (0.00–2.00)		1.12 ± 0.40	1.00 (0.00–2.00)		0.609
	1 year	0.67 ± 0.37	0.75 (0.00–1.00)		0.45 ± 0.43	0.25 (0.00–1.00)		
	3 years	0.59 ± 0.50	0.50 (0.00–2.00)		0.52 ± 0.45	0.25 (0.00–1.50)		
Gingival index	Baseline	1.11 ± 0.23	1.00 (0.00–2.00)		1.08 ± 0.27	1.00 (0.00–2.00)		0.356
	1 year	0.36 ± 0.45	0.00 (0.00–1.00)		0.13 ± 0.29	0.00 (0.00–1.00)		
	3 years	0.37 ± 0.54	0.00 (0.00–2.00)		0.32 ± 0.49	0.00 (0.00–2.00)		
Bleeding on probing (%)	Baseline	97.12 ± 12.8	100.00 (50.00–100.00)		97.12 ± 8.15	100.00 (75.00–100.00)		0.969
	1 year	35.58 ± 30.14	25.00 (0.00–100.00)		29.81 ± 30.02	25.00 (0.00–100.00)		
	3 years	40.38 ± 33.22	50.00 (0.00–100.00)		35.58 ± 33.30	25.00 (0.00–100.00)		
Probing depth (mm)	Baseline	5.90 ± 1.42	5.88 (3.75–8.75)	T 1–2 < 0.001	5.41 ± 1.16	5.25 (3.75–8.25)	T 1–2 < 0.001	0.007
	1 year	3.71 ± 1.09	3.50 (2.00–6.00)	T 1–3 < 0.001	2.70 ± 0.80	2.50 (1.50–5.25)	T 1–3 < 0.001	
	3 years	3.80 ± 1.41	3.50 (2.00–8.00)	T 2–3 0.582	3.28 ± 1.27	3.00 (2.00–7.00)	T 2–3 0.005	
Clinical attachment level (mm)	Baseline	5.93 ± 1.34	5.88 (3.75–8.25)		5.47 ± 1.31	5.25 (3.75–8.25)		0.156
	1 year	3.98 ± 1.22	3.63 (2.00–6.00)		2.92 ± 1.00	2.50 (1.50–5.25)		
	3 years	4.23 ± 1.55	3.75 (2.25–9.00)		3.66 ± 1.67	3.25 (2.25–9.00)		
Mucosal recession (mm)	Baseline	0.04 ± 0.20	0.00 (0.00–1.00)		0.06 ± 0.20	0.00 (0.00–0.75)		0.313
	1 year	0.25 ± 0.39	0.00 (0.00–1.00)		0.27 ± 0.44	0.00 (0.00–1.50)		
	3 years	0.38 ± 0.48	0.25 (0.00–2.00)		0.46 ± 0.55	0.25 (0.00–2.00)		
Radiographic vertical defect depth (mm)	Baseline	4.10 ± 1.56	4.20 (1.90–7.15)	T 1–2 < 0.001	3.66 ± 1.02	3.38 (1.95–6.32)	T 1–2 < 0.001	<0.001
	1 year	2.51 ± 1.45	2.14 (0.50–5.50)	T 1–3 < 0.001	1.67 ± 0.76	1.03 (0.42–3.11)	T 1–3 < 0.001	
	3 years	2.76 ± 1.56	2.35 (0.50–5.98)	T 2–3 0.066	1.99 ± 0.76	1.49 (0.43–5.12)	T 2–3 0.002	

^*^, General linear mixed model (multiple comparisons by group) Bonferroni-Dunn test, *p* < 0.05 considered statistically significant

^†^, Repeated measures of analysis of variance, *p* < 0.05 considered statistically significant

*CGF*, concentrated growth factor; *CM*, collagen membrane. *T1*, baseline; *T2*, 1 year; *T3*, 3 years

**Fig. 4:**
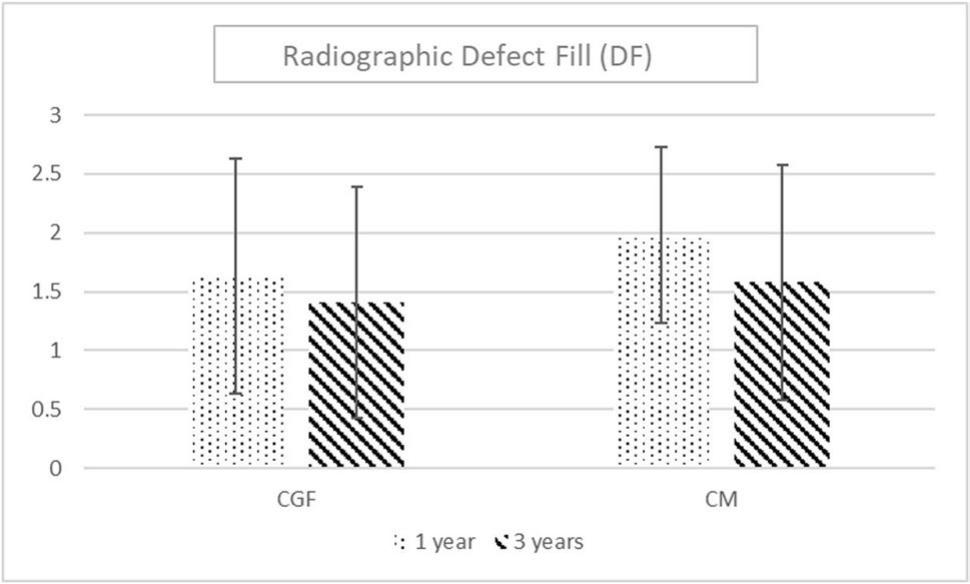
Comparison of the defect fill between the groups. Mann–Whitney test, *p* < 0.05 considered statistically significant

In the CGF group, the mean VDD significantly decreased from 4.15 ± 1.44 to 2.51 ± 1.45 mm at year 1, and to 2.76 ± 1.56 mm at year 3, while in the CM group, it significantly dropped from 3.66 ± 1.02 to 1.67 ± 0.76 mm at year 1, and to 1.99 ± 0.76 mm at year 3. Comparing the trends of decrease for the study groups, a statistically significant difference was observed between the groups in favor of the CM group (*p* < 0.001). A trend of increase was observed at year 3 compared to year 1 postoperatively for both groups and the trend towards a significantly higher increment was found between the time intervals in the CM group (*p* = 0.002), whereas the increment did not reveal statistical significance in the CGF group (*p* = 0.066). The radiographic evidence of DF between baseline and year 1 and year 3 after the reconstructive surgical therapy were 1.63 ± 1.00 mm and 1.41 ± 0.98 mm in the CGF group and 1.98 ± 0.75 mm and 1.58 ± 1.00 mm in the CM group, showing no statistically significant differences between the groups (Fig. 4).

**Fig. 5:**
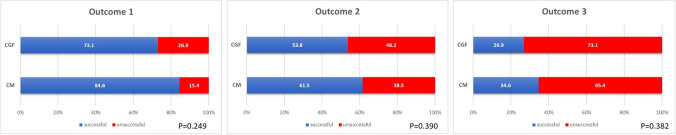
Comparison of the treatment outcomes at 3 years postoperatively. Independent Student’s t-test, *p* < 0.05 considered statistically significant

As to the composite outcomes, for outcome 1, 73.1% and 84.6% of the implants were considered successful in the CGF and CM groups at year 3 after reconstructive surgical therapy. For outcome 2, treatment was successful for 53.8% and 61.5% of the implants in the CGF and CM groups, and for 26.9% and 34.6% of the CGF and CM groups that did not show any BOP or Supp in addition to outcome 2 (outcome 3). None of the composite outcomes revealed any statistically significant difference between the groups (Fig. 5).

When evaluating the effects of implant- or patient-related variables on composite outcomes, no significant impact was found on outcome 1 or outcome 3. While the present study did not identify an association between the variables tested in multilevel analyses and treatment outcomes, results of univariate analyses with the outcome 2 as the dependent parameter demonstrated that history of periodontitis, baseline VDD values, and the number of residual intrabony defect walls were significantly associated with outcome 2. According to the analysis, both the presence of history of periodontitis and baseline VDD values were correlated with a statistically significant negative effect on outcome 2 (*p* = 0.033; OR = 3.50 and *p* = 0.039; OR:0.975, respectively). Regarding the peri-implant bone defect configurations, four-wall defects showed 6.0 and 7.0 times higher odds ratios for the success of the treatment outcome compared to three- and two-wall defects, respectively (Table 3).

**Table 3:** Multilevel analyses assessing possible predictive indicators on the 3-year treatment success (outcome 2)

Variables	β	S.E	*p* value	OR	95% CI	
					Lower bound	Upper bound
Treatment method	0.316	0.563	0.575	1.371	0.455	4.136
Gender	0.773	0.572	0.176	2.167	0.706	6.645
Age	-0.024	0.032	0.443	0.976	0.917	1.039
History of periodontitis	-1.253	0.589	0.033*	3.500	1.104	11.094
Smoking (< 10 cigarettes a day)	1.019	0.630	0.106	2.769	0.806	9.512
Implant location (maxilla-mandible)	0.038	0.572	0.947	1.038	0.339	3.185
Implant location (anterior–posterior)	1.135	0.917	0.216	3.111	0.515	18.778
Implant function time (year)	-0.104	0.131	0.427	0.901	0.696	1.165
Baseline probing depth (mm)	-0.264	0.235	0.260	0.768	0.484	1.217
Baseline bleeding on probing (%)	-0.013	0.032	0.689	0.987	0.928	1.051
Baseline clinical attachment level (mm)	-0.335	0.250	0.180	0.715	0.439	1.167
Baseline radiographic vertical defect depth (mm)	-0.488	0.236	0.039*	0.614	0.386	0.975
Number of bone walls (four-wall) two-wall defects	1.946	0.862	0.024*	7.000	1.293	37.909
three-wall defects	1.792	0.764	0.019*	6.000	1.343	26.808

*β,* beta coefficient; *S.E*., SE of estimate; *CI*, Confidence interval

## Discussion

The present study focused on the evaluation of 3-year outcomes of an RCT on reconstructive surgical treatment of peri-implantitis using a bone substitute combined with CGF or CM. It was identified that reconstruction of peri-implant vertical bone defects by means of a grafting material with CM yielded better results compared to using CGF at year 3 after surgical intervention. The changes in PD between baseline and year 1 and baseline and year 3 presented significantly greater decreases for the CM group in comparison with the CGF group, although a significantly higher increase between year 1 and year 3 was shown for the CGF group. The present study also revealed no significant differences between the two treatment modalities in terms of treatment success outcomes. Furthermore, the presence of a history of periodontitis, baseline VDD values, and the number of residual intrabony defect walls were found to be prognostic indicators significantly associated with the combined outcome variables with a PD of ≤ 5 mm and the absence of additional bone loss at year 3 postoperatively.

Over the years, it has been proven that surgical augmentative therapies demonstrated promising results in terms of reconstruction of peri-implant bone defects, achieving re-osseointegration, and limiting peri-implant MR [10]. The materials and combinations of materials would present better outcomes by means of reconstructive surgical approaches that have also been widely debated. To the present date, many studies in which bovine-derived xenografts with or without a barrier membrane were used for the reconstruction of peri-implant bone defects exhibited a PD reduction range of 1.7–3.8 mm [11, 13, 25–28] and radiographic vertical defect reduction range of 0.7–3.0 mm [11, 25, 27–29] at year 1 postoperatively. The discrepancies regarding clinical and radiographic outcomes might probably be explained by using or not using a barrier membrane or another biological agent, different baseline bone defects characteristics, different implant surface characteristics, and using different implant surface decontamination methods.

On the other hand, there is a limited supply of data available for evaluating the long-term success of reconstructive surgical therapies and more information is needed about the assessment of reconstructive modalities over a longer period of time. A long-term follow-up study by Rocuzzo et al. (2020) [30] investigated the 10-year outcomes following a reconstructive treatment of peri-implantitis using deproteinized bovine bone mineral with 10% collagen in two different implant surface characteristics (SLA vs. TPA). The proposed reconstructive treatment exhibited stable clinical and radiographic outcomes, especially for SLA implants, despite the fact that a tendency to relapse was observed in long term (7 and 10 years). On the other hand, a recent follow-up observation reporting the 5-year results of reconstructive management by means of mineralized dehydrated bone allograft in combination with resorbable membrane was also demonstrated increased PD and a progressive decrease in vertical defect filling although favorable short-term results were presented [9]. The present results were comparable with the aforementioned studies in that an overall improvement was observed for both study groups at year 1 postoperatively. The mean PD dropped by 2.21 (1.43) mm in the CGF group, and by 2.60 (1.00) mm in the CM group, and radiographic DF was found to be 1.63 (1.00) mm and 1.98 (0.75) mm. However, a trend towards a slight decrease was identified for the mean PD reduction (2.03 mm with a SD of 1.72 mm, and 2.12 mm with a SD of 1.52 mm) and radiographic DF (1.41 mm with a SD of 0.98 mm, and 1.58 mm with a SD of 1.00 mm) in the CGF and CM groups at 3-year follow-up. Consistent with the present finding, Khoskam et al. (2016) stated that the amount of radiographic vertical defect filling ranged from 1.46 to 3.30 mm after 3 years of healing by reconstructive therapies of peri-implantitis in their systematic review [31].

Using a barrier membrane over the grafting material, which is the principle of guided bone regeneration (GBR), is strictly recommended based on the theoretical understanding that it prevents the soft tissue ingrowth into a defect region, allows the angiogenic and osteogenic cells to migrate into the blood clot, and provides stabilization of the grafting material in the defect [32]. Nonetheless, considering the clinical outcomes obtained from the related studies, the benefits of barrier membranes in the augmentative surgical treatment of peri-implantitis have not been clarified. While a systematic review and meta-analysis indicated that additional use of barrier membranes in the surgical treatment of peri-implantitis resulted in higher reductions of PD and BOP compared to bone substitutes alone [33], a 5-year follow-up of a randomized controlled clinical study exhibited no additional benefits to using barrier membranes and also proved a cost-increasing procedure [13]. In a Consensus Report of Group 4 of the 15th European Workshop on Periodontology, it was highlighted that reconstructive treatment of peri-implant defects by xenogeneic material combined with a membrane showed greater BOP and PD reductions as well as CAL gains [9]. On the other hand, a recent RCT demonstrated approximately 3 mm of radiographic defect reduction by means of bovine-derived xenografts alone in a reconstructive surgery of peri-implant defects, which was comparable to the findings of other combined approaches of reconstructive treatments [25]. However, limited success in the resolution of inflammation was also reported in that study, which may be explained by not using a barrier membrane.

Autologous blood concentrates have been successfully used in alveolar bone regeneration before implant placement and reconstruction of peri-implant defects in the course of implant placement or in peri-implantitis augmentative therapy, improving soft tissue wound healing/regeneration [34]. However, it must be mentioned that limited evidence is available on the impact of platelet concentrates on clinical outcomes of reconstructive surgical therapy of peri-implantitis. It has been stated that platelet membranes act as a cleansing barrier that would be dissolved, allowing bone growth onto the decontaminated implant surface in peri-implantitis cases [35]. Besides, a recent in vitro study also exhibited that L-PRF membranes significantly reduce bacterial counts on biofilm-infected rough implant surfaces through the release of antimicrobial peptides by platelets inducing osmotic death of the bacteria [35]. Therefore, the effects of platelet concentrates on peri-implantitis in clinical setting should also be explored in the context of microbiological outcomes in addition to clinical and radiographic outcomes. On the other hand, platelet membranes are resorbed within 10 to 28 days, which may limit their use as a resorbable barrier membrane because they could barely maintain a sufficient space for reconstruction of bone defects [36]. Besides, a recent systematic review and meta-analysis has also marked that the use of platelet concentrates has yielded a favorable soft tissue healing compared to hard tissue healing [37]. However, it has been emphasized that success in bone regeneration might also be linked with anti-infectious action and immune regulation of the leukocytes and neutrophils clustered in the fibrin clot as notably earlier soft tissue healing was observed after the administration of A-PRF and CGF membranes [16]. Coherently, the present study revealed stable clinical and radiographic parameters following reconstructive surgical therapy where CGF membranes were used for an observation period of three years. It is also worth noting that the CGF group did not exhibit a remarkable change in peri-implant soft tissue level at follow-up examinations in what was suggested as one of the critical outcomes after reconstructive therapy of peri-implantitis [9]. This finding could also be explained by the impact of centrifugation protocol on the cells (i.e., leukocytes, platelets, lymphocytes, and monocytes), growth factors, and fibrin architecture of CGF, which are significantly associated with soft and hard tissue healing. Slower centrifugation speed [38] or horizontal centrifugation [39] has exhibited promising results in terms of releasing a greater amount of growth factors and having a higher concentrations of platelets and leukocytes in the matrix. In the present study, the centrifugation protocol was applied according to the manufacturer’s instructions using fixed-angle centrifugation.

In the reconstructive surgical approaches of peri-implantitis treatment, a promising concept is submerged healing which is based on the understanding of aseptic healing of bone substitute and barrier membrane in a protective environment in GBR procedures. This approach has been recommended for the fact that submerged post-operative wound closure allows to potentially adapt for better oral hygiene ability [5] and provides spontaneous augmentation of the keratinized mucosa on the crestal area [40]. Although no evidence-based superiority of submerge has been demonstrated on the healing site regarding the outcome in comparison with non-submerged healing [41], reconstructive approach in submerged manner has been documented to yield optimal clinical and radiographic outcomes in terms of disease resolution and PD reduction and radiographic defect fill [13, 40].

It is important to note that peri-implant maintenance programs play a critical role in the resolution of inflammation in addition to treatment success following peri-implantitis surgery [42]. In particular, the stability of clinical outcomes of the reconstructive surgical approaches to peri-implantitis is dependent on optimal plaque control performed by the patient and compliance with a regular maintenance program, similar to regenerative periodontal treatment [43]. A previous study by Serino, Turri, and Lang (2015) demonstrated that no recurrence of peri-implant disease was observed around any of the implants that regained healthy peri-implant conditions following peri-implant surgery for the patients attending SPIT during the 5-year follow-up period [44]. Moreover, that study also indicated that stable peri-implant conditions were maintained in the majority of the implants (61%) with one or two residual pockets (4–5 mm or ≥ 6 mm) and only a few implants (13%) showed disease progression after the surgery. A recent 5-year follow-up study by Heitz-Mayfield et al. (2018) also evaluated the clinical outcomes of SPIT following surgical therapy of peri-implantitis and pointed out stable peri-implant conditions in the majority of implants in two-thirds of the patients after the surgery [45]. They reported that residual pockets (PD ≥ 5 mm with concomitant BOP/Supp) were present in 7 (19%) out of the 36 treated implants 12 months after the treatment, and that four implants (11%) of four patients were lost during the 5-year period [45]. In the present study, residual pockets (PD > 5 mm with concomitant BOP/Supp) were observed in 10 patients (40%) of the CGF group and 7 patients (26.9%) of the CM group at the 3-year follow-up period. In terms of the residual pockets’ concomitant with increased VDD presenting the recurrence of peri-implantitis, out of initial 52 treated implants, 15 implants (28.8%) exhibited peri-implantitis during the 3-year period. In a recent study, Carcuac et al. (2020) have reported a higher frequency of peri-implantitis recurrence/progression (44%) 5 years after surgical therapy of peri-implantitis than those demonstrated in the present study [46]. This difference may be related to the longer follow-up period of that study.

The use of composite therapeutic outcomes has been proposed to evaluate the effectiveness of interventions to treat peri-implantitis [19]. Multiple studies have reported the efficacy of treatment procedures based on different success criteria [24, 25, 29, 30, 45, 47]. In the present study, three different composite outcomes were assessed to determine treatment success based on the absence of additional radiographic bone loss in combination with clinical landmarks (PD with concomitant BOP and Supp). In a recent RCT, no additional bone loss following the treatment has been suggested to remark healthy peri-implant tissues during the follow-up period [24]. The percentage of the positive predictive value of BOP alone for peri-implantitis diagnosis has been found highly variable (7–60%) and it is indicated that the positive predictive value of BOP measurements might be low [48]. On the other hand, according to the consensus report of Workgroup 4 of the 2017 World Workshop, it can be difficult to define a range of PD values compatible with the differentiation of peri-implant health and disease [1]. In line with these reports, it should be noted that these assessments might be misleading in identifying peri-implant health. Therefore, in this study, different composite outcomes were created with different combinations of the success criteria. Treatment success did not significantly differ among different groups in terms of all of the defined criteria. The outcomes were acquired for 73.1%, 53.8%, and 26.9% in the CGF group, and 84.6%, 61.5%, and 34.6% in the CM group for outcomes 1, 2, and 3, respectively. Recent systemic reviews have identified that the success rate ranged between 11 and 66.7% of the implants and 42.3% of the patients in a 1–7 years after reconstructive surgical approaches to peri-implantitis [4, 8]. It has also been reported that this variety might probably be explained by using different treatment success criteria evaluating different surgical modalities and the diversity in the surface characteristics of treated implants [4].

Whereas the present study did not reveal an association between the implant- or patient-related variables tested in multilevel analyses and outcomes 1 and 3, history of periodontitis, baseline VDD values, and number of bone defect walls were identified as statistically significant variables affecting outcome 2. It means that these prognostic indicators significantly influenced the treatment success defined by a maximum PD of ≤ 5 mm combined with no additional bone loss, whereas no significant relationship was identified with the absence of additional bone loss alone. This finding highlighted that history of periodontitis and bone defect configurations and vertical extent could have an impact on residual PD around implants after reconstructive therapy of peri-implantitis. In accordance with the present finding, the efficacy of defect bone configurations on the mean value of PD changes between baseline and after reconstructive treatment has been recently demonstrated in a study by Aghazadeh et al. (2020) [49], who reported that four-wall defects exhibited significantly higher PD reductions compared to two- or three-wall defects. On the other hand, there is strong evidence that patients with a history of periodontitis have an increased risk for peri-implantitis [1]. It is worthy to underline that history of periodontitis may have a significant influence on soft and hard tissue morphology around the implants as well as serving like reservoirs for pathogens and therefore may pose an impact on residual peri-implant pockets after therapy of peri-implantitis. The current data identified a history of periodontitis as a prognostic indicator, which is associated with a negative treatment outcome. However, a previous study evaluating prognostic factors for surgical treatment outcome of peri-implantitis by de Waal et al. (2016) could not demonstrate the history of periodontitis as a significant predictor [50]. This finding could possibly be related to the fact that a significant proportion of the patients included in that study were completely edentulous and the data on the history of periodontitis were mainly based on self-reported information. In the current study, it was shown that the amounts of VDD values at baseline were negatively associated with the treatment outcome in agreement with the findings of previous studies that observed peri-implant bone loss at baseline as a negative predictive factor for the success of treatment outcome [50–52]. Besides, studies have also exhibited that peri-implant bone defect configuration has a potential impact on the outcome of reconstructive therapy of peri-implantitis [49, 51, 53]. It has also been highlighted in a recent review by Schwarz et al. (2022) that augmentative treatment outcomes were affected by the morphology of the peri-implant bone defects [8]. The present study identified a favorable treatment outcome with four-wall defects compared to the other defect types in reconstructive surgical therapy of peri-implantitis consistent with a recent study conducted by Aghazadeh et al. (2020) [49].

The present study had several limitations. One of them was the lack of the assessment of the effect of implant surface characteristics on the success of treatment modalities at year 3 following the operation. The implants included in the present study had modified and non-modified surfaces, depicting no significant difference between the study groups. On the other hand, surface characteristics could not be found as a prognostic indicator in the multilevel analyses performed in the RCT where the 1-year results were evaluated [18]. Second, the influence of the present treatment modalities could also be investigated on the dimensions of keratinized mucosa that has been proposed to have a critical role in maintaining peri-implant health. Lastly, a comparison of the effectiveness of the present treatment modalities is needed to identify a longer follow-up presenting at least 5 years.

## Conclusion

Reconstructive surgical therapy of peri-implantitis using a bone substitute combined with CGF and CM resulted in stable clinical and radiographic parameters from 1 to 3 years postoperatively in cases on periodontal/peri-implant maintenance care. The changes in the mean PD and VDD between baseline and 1 year and baseline and 3 years exhibited significantly higher reductions for the CM group compared to the CGF group. The present study also revealed no significant difference between the two treatment modalities in terms of treatment success outcomes. The success of the treatment outcome was negatively affected by identified prognostic indicators, i.e., history of periodontitis, VDD values at baseline, and peri-implant bone defect configurations. Four-wall bone defects seemed to yield a better success compared to the other defect types in reconstructive surgical therapy of peri-implantitis.
